# Primary evaluation of an air-cooling device to reduce oral mucositis: a pilot study in healthy volunteers

**DOI:** 10.1007/s12032-020-01431-4

**Published:** 2020-11-10

**Authors:** C. Blacker, T. Kamsvåg, R. S. Bejhed, G. Ljungman

**Affiliations:** Department of Women’s and Children’s Health, Uppsala University, Entrance 95, SE-751 85 Uppsala, Sweden

**Keywords:** Oral mucositis, Oral cryotherapy, Intra-oral air-cooling device, Innovative medical technology

## Abstract

Oral mucositis is a common side effect of chemo and radiotherapy causing painful ulcers in the oral mucosa. One of the preventive treatments recommended in international guidelines is oral cryotherapy (OC). Randomized clinical trials on OC have used ice and ice-chips to cool the mouth, but this cooling method can be difficult for the patients to tolerate. Studies have shown that OC with ice for a period of 60 min reduces the oral temperature by 12.9 °C. The aim of this pilot study was to evaluate the temperature reduction and tolerability of OC using an intra-oral air-cooling (IOAC) device in healthy volunteers. Twelve healthy volunteers, mean age 35.4 years, were included in the study. They were treated with OC using the IOAC device for 60 min. Measurements of temperature were obtained at baseline, 5 and 60 min using a FLIR® C2 camera. After the OC session, tolerability and adverse events were documented using a questionnaire. All participants were able to use the device for 60 min. The overall temperature reduction after 5 min of OC was 10.7°C (*p* < 0.01) and after 60 min 14.5°C (*p* < 0.01). The most common adverse events were bad fit of the mouthpiece (*n* = 6), hypersalivation (*n* = 6), and difficulties swallowing (*n* = 5). The oral device reduced the temperature of the oral mucosa as much as treatment with ice with tolerable adverse events. The mouthpiece will be remodeled to improve tolerability before further studies are conducted.

**Electronic supplementary material:**

The online version of this article (10.1007/s12032-020-01431-4) contains supplementary material, which is available to authorized users.

## Introduction

Oral mucositis (OM) is a common side effect of chemo and radiotherapy in both children and adults [1–3]. OM is considered one of the most debilitating side effects of chemotherapy treatments according to patients, and it causes pain, nutritional difficulties and an increased risk of infections [4–7]. OM can be a dose-limiting factor leading to reduced doses and postponed treatments thus affecting the prognosis of the patient [1]. Among patients receiving standard chemotherapy regimens, the incidence of OM is around 40%; for patients receiving radiotherapy to the head and neck, the incidence is close to 100%; and in patients undergoing hematopoietic stem cell transplantations, the incidence is around 80% [1, 8]. The risk of developing OM varies depending on the treatment regimen and doses as well as personal risk factors including genetic factors, age, smoking, and previous episodes of OM [9–11].

There are no conclusive recommendations on how to prevent OM in patients. Preventive treatments recommended in international guidelines are oral cryotherapy (OC), recombined human keratinocyte growth factor and low-level laser therapy [12]. OC, the cooling of the mouth with ice during chemotherapy infusions, is a cost-effective and safe treatment with few adverse events and is recommended for patients receiving chemotherapy with short half-lives such as 5-FU or high-dose melphalan prior to a hematopoietic stem cell transplantation [13, 14].

The mechanism of how OC prevents OM is not fully understood. The hypotheses are that the cooling causes a vasoconstriction in the tissue and a lower metabolism of the basal epithelial cells resulting in lower toxicity and hence less tissue damage [15, 16]. In randomized clinical trials, cooling has been achieved using ice and ice-chips for 30-60 min during chemotherapy infusions [15, 17, 18]. Studies have shown that OC with ice for 60 min reduces the temperature in the oral mucosa by 8.1–12.9 °C [19, 20]. However, the intervention is experienced as troublesome for many of the patients. Reported adverse events are teeth tingling, freezing, headache, and nausea [19, 20]. For children, compliance to OC is even more problematic. Among 26 children using OC only 58% were able to use it for 30 min [21].

The aim of this study was to investigate a new technique to cool the mouth, using an IOAC device, by evaluating the temperature reduction of the mouth, tolerability, and adverse events.

## Subjects and methods

### Participants

Twelve healthy volunteers were recruited among a convenience sample of colleagues and individuals from our professional networks. Eleven participants were men and the average age was 35.4 years (SD 6.2) (range 25–43). They all reported to be healthy and were not taking any medication. Of the participants, three reported never having sensitive teeth, seven rarely, and two that they sometimes had sensitive teeth.

### The air-cooling device

The device consists of a cooling unit, a cooling delivery system, and a mouthpiece. The cooling unit is an insulated container that holds three piezo-electric cooling elements connected in series, constantly cooling the air. Room temperature air is blown through the system using a motor-powered fan, and the cooled air is monitored by airflow and temperature sensors. The cooling delivery system is a set of insulated tubes connecting the cooling unit with the mouthpiece. The mouthpiece is a 3D printed biocompatible plastic device in the size to fit an adult but can be tailored to fit any size. The cooling device is connected to a computer for data collection. The average temperature of the cooled air after 60 min was 3.4°, measured at the mouthpiece.

### Methods

The measurements of temperature were obtained using a digital thermal imaging camera. All participants were placed sitting in the same position at 25 cm from the camera. The camera used was a FLIR® C2 that has a thermal minimal focus distance of 0.15m, comparative accuracy of 0.01°C, thermal sensitivity of <0.10°C, and a penetration of 4.5–6.5 mm (spectral range 7.5–14 µm) of the outer mucosal surface [22, 23]. The images created are standard JPEG images in a selectable color setting (Iron, Rainbow, Rainbow HC, Gray). Using the rainbow color setting, the highest temperatures in the images are presented in red and the lowest in black. Images were downloaded to a computer using the FLIR tools + software (Version 5.13.18031.2002) for data analysis.

### Experimental procedure

The study was approved by the Regional Ethical Committee in Uppsala, Sweden (Regionala Etikprövningsnämnden Uppsala, Dnr 2018/326). Before participating in the study, each participant received written and verbal information about the study, and written informed consent was obtained.

The participants were placed in a room, with an average temperature of 22.4 °C, for 30 min for acclimatization before the experiment started. Baseline images of the oral mucosa were obtained of the right and left buccal mucosa, upper and lower lips, anterior, posterior and ventral aspects of the tongue and hard palate by the first author acting as examiner. The experiment was initiated with a 5-min test phase to test the IOAC device. Images of the above-mentioned areas were taken after 5 min, after which the experiment continued for the period of 60 min and a new set of images was collected. Participants were able to interrupt the experiment at any time if they wanted. Tolerability was defined as being able to use the device for ≥ 90% of the 60 min. After completing the experiment, the participants were asked to answer a questionnaire about tolerability and adverse events (appendix 1).

During the experiment, data regarding output temperature and flow were continuously fed from the IOAC device to a computer. In order to gain consistency of raw data, all images were recorded by the first author. Analysis of the images was done by the two first authors together in agreement.

### Statistics

**Fig. 1:**
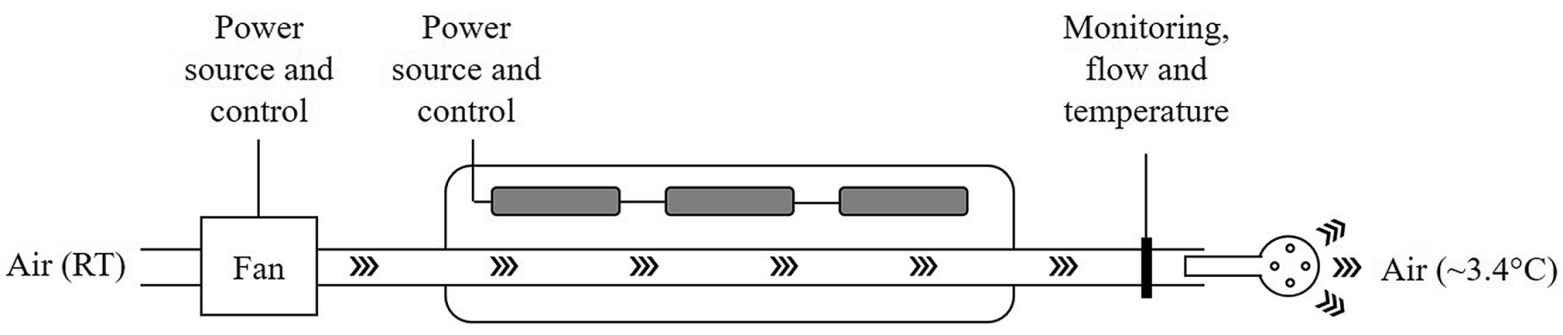
Schematic drawing of cooling device. The average air temperature after cooling for 60 min was 3.4°C. RT = room temperature

The temperature reduction data were controlled for normal distribution with the Kolmogorov-Smirnov test. Data were normally distributed, and therefore, we used the parametric paired t test. Tolerability and adverse events were reported using descriptive statistics. A *p* value < 0.05 was considered statistically significant. The analysis was done using SPSS version 26 statistical analysis package (IBM, Armonk, NY, USA) (Fig. 1).

## Results

All participants were able to complete the OC session of 60 min. The mean temperature reduction was 10.7 °C (*p* < 0.01) after 5 min and 14.5 °C (*p* < 0.01) after 60 min (Table 1).

**Table 1:** Mean temperatures in the oral mucosa at baseline, 5 min, 60 min, and the mean temperature differences

	Baseline T ± SD	5 min T± SD	Δ T, 5 min	*p* value	60 min T ± SD	Δ T, 60 min	*p* value
All surfaces	35.0 ± 0.6	24.3 ± 1.6	10.7	< 0.01	20.5 ± 1.3	14.5	< 0.01
Tongue dorsal anterior	34.3 ± 1.2	22.3 ± 4.1	12.0	< 0.01	18.0 ± 2.1	16.3	< 0.01
Tongue dorsal posterior	34.5 ± 1.1	20.3 ± 2.8	14.2	< 0.01	16.3 ± 1.8	18.2	< 0.01
Tongue dorsal ventral	36.0 ± 0.6	26.8 ± 2.1	9.2	< 0.01	24.1 ± 2.5	12.0	< 0.01
Buccal mucosa right	36.0 ± 0.7	24.2 ± 2.6	11.8	< 0.01	20.4 ± 1.8	15.6	< 0.01
Buccal mucosa left	36.1 ± 0.5	24.3 ± 2.0	11.8	< 0.01	20.5 ± 1.9	15.6	< 0.01
Labial mucosa upper	34.1 ± 1.0	24.7 ± 2.2	9.4	< 0.01	21.1 ± 3.1	12.9	< 0.01
Labial mucosa lower	34.0 ± 1.4	28.9 ± 2.9	5.1	< 0.01	24.8 ± 3.8	9.2	< 0.01
Hard palate	34.7 ± 0.9	22.7 ± 3.2	12.0	< 0.01	18.8 ± 2.2	15.9	< 0.01

Paired *t* test. *T* temperature in °C, *SD* standard deviation, *Δ T* temperature difference in °C

**Fig. 2:**
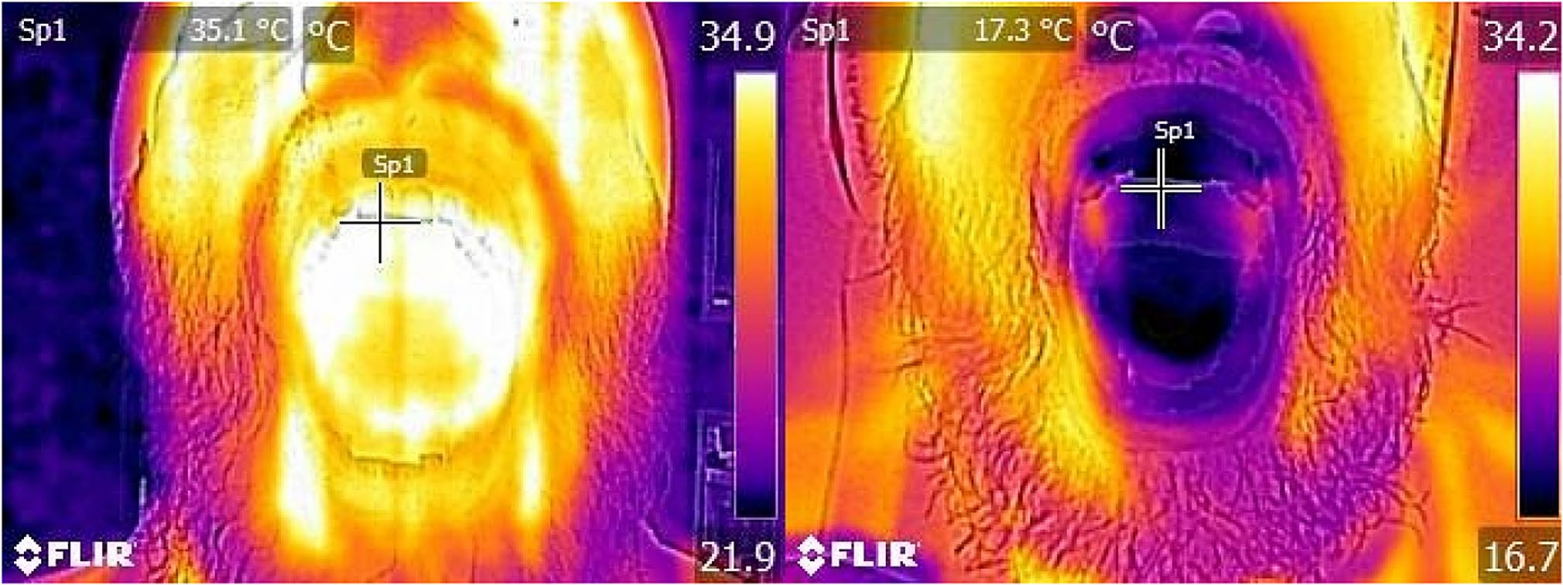
Digital thermal image of a test subject. Left before OC; right after 60 min OC

**Fig. 3:**
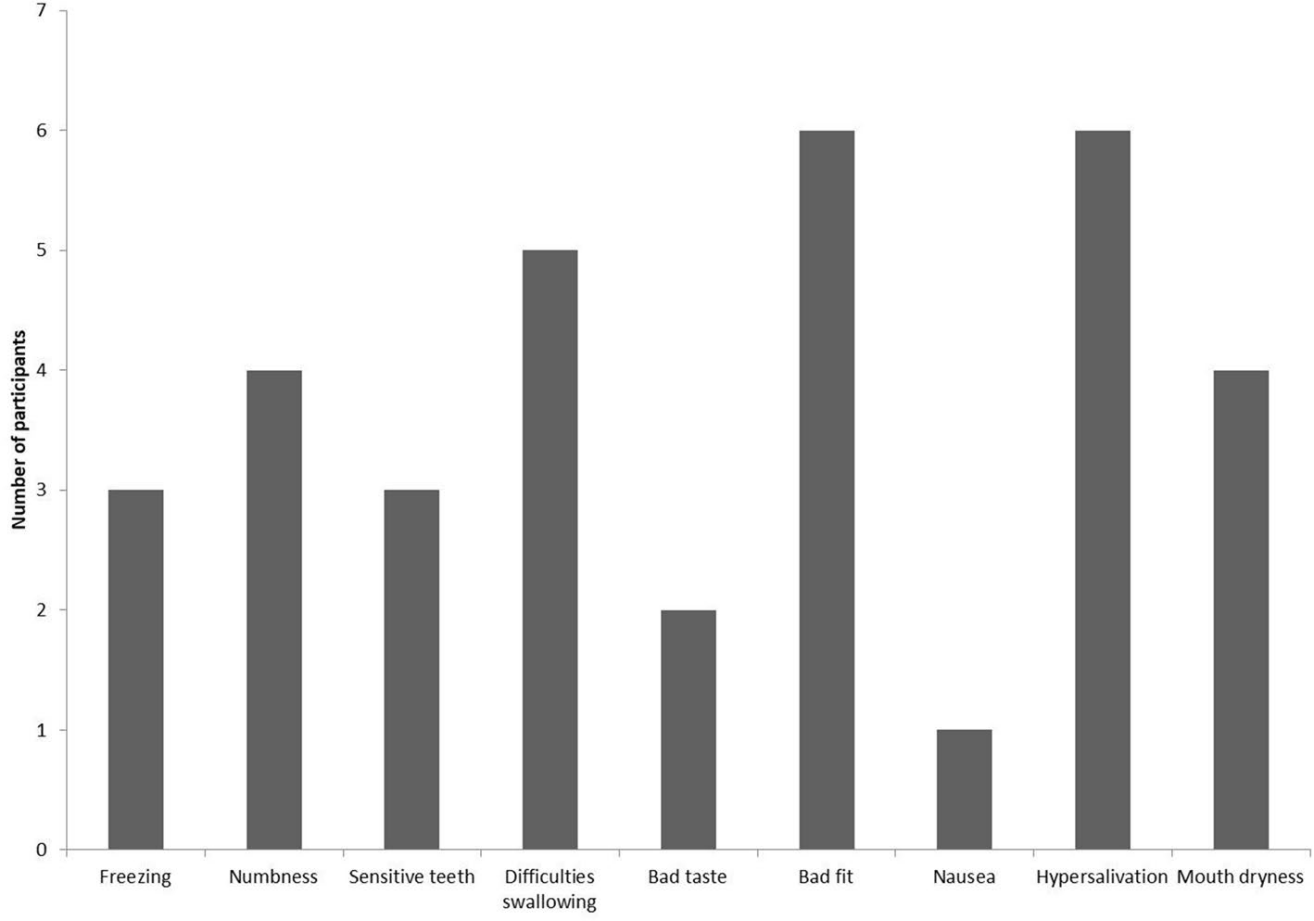
Reported adverse events. More than one option possible for every participant

One participant experienced the intervention as very unpleasant, four as a little unpleasant, five not really unpleasant, and two as not unpleasant at all. The reported adverse events are presented in Fig. 2. Three participants had difficulties keeping the IOAC device in their mouths for the first 5 min, two participants had to take short breaks during the intervention, but all participants were able to use the device for ≥ 90% of the time (Fig. 3).

All participants answered that they would be able to comply with the therapy in the fictive scenario that they were to undergo chemotherapy treatment and that the treatment could prevent painful ulcers in the mouth.

### Device outputs

The mean output temperature after 5 min was 4.0 °C and after 60 min 3.4 °C. The mean airflow was 48 standard liters per minute (slm) after five and 60 min. In seven of the sessions, one of the three cooling elements malfunctioned, resulting in loss of cooling capacity from that specific element. However, this did not affect the output temperature. In two sessions, the insulation of the tube between the cooling unit and mouthpiece was displaced resulting in higher output temperatures (mean 6.9 °C at 5 min and 6.8 at 60 min). This resulted in a lower temperature reduction at 60 min for these two participants compared to the rest (13.1 °C compared to 14.7 °C, *p* = 0.03).

## Discussion

OM is a debilitating condition and today’s standard of treatment results in insufficient compliance. This presents a need to explore new potential solutions and treatment strategies targeting OM. In our study, we demonstrated that cold air is capable of reducing the average oral mucosal temperature by 14.7 °C after 60 min of treatment with an average temperature of 3.4 °C. We also demonstrated that cold air is capable of reducing the temperature in the oral mucosa by 10.9 °C after just 5 min of treatment with an average temperature of 4.0 °C. These results are comparable to those of Walladbegi et al. [20] and Svanberg et al. [19] who demonstrated that the cooling of the oral mucosa with ice after 60 min of treatment in healthy volunteers led to a temperature reduction of 8.1 °C and 12.9 °C, respectively.

All participants were able to use the device for > 90% of the time. Similar results were obtained by Svanberg et al. [19], and tolerability was better compared to Walladbegi et al. [20], indicating this new IOAC device has similar or higher acceptance level compared to ice treatment in healthy volunteers. An explanation for a lower tolerability with ice could be that it is more difficult to use ice for OC for a prolonged period of time. We reported similar results of adverse events to those presented by Walladbegi et al. [20], but with a difference in incidence of the various adverse events such as lower incidence rates of being cold, feeling of numbness, sensitive teeth, and headache. This suggests that the adverse events related to the actual cooling process of the oral mucosa were lower with the IOAC device compared to ice. This could possibly be explained by higher temperatures in the air compared to ice. Also we had lower incidences of nausea and bad taste. On the other hand, we reported higher incidence of difficulties swallowing, increased salivation, and tension in the jaw musculature. Difficulties swallowing seemed to be related to having the IOAC device in the mouth while trying to swallow. In addition, bad fit of the IOAC device was sometimes a problem in our study. Bad fit included the IOAC device being difficult to hold in the mouth because we did not have a supporting structure, resulting in tension of the jaw musculature and fatigue, and the insulating tubes between the cooling unite and IOAC device was too short, making the device uncomfortable to use. These are design problems that require alterations in the next prototype.

Another interesting aspect that has not been studied previously is whether participants believed that using cold air as a modality for cryotherapy for prevention of oral mucositis could be a feasible option. All participants agreed to this statement.

One strength of this study is the experimental environment for the study and retrieval of data. This allowed us to measure factors such as environmental temperature, device output temperature and flow, and a standardization of data collection. Further strengths include the investigation of a limited number of important parameters avoiding mass significance.

Limitations of this study include a small population of which all were healthy volunteers recruited from colleagues and individuals in our professional network, which could result in highly motivated and resilient individuals. There were also complications with the IOAC device. One of the cooling elements malfunctioned for seven of 12 participants, but this did not affect the cooling temperature output of the cooling device, indicating that the cooling device had an overcapacity to cool air sufficiently. Further malfunctions were the displacement of insulating parts of the cooling device, resulting in higher output temperatures from the cooling device. It could be argued that these machine malfunctions resulted in decreased cooling ability.

This study has demonstrated that a new method of OC is plausible with fewer reported adverse effects, despite adding new adverse events, compared to OC with ice. The new method could possibly open up for more patient-specific OC strategies and treatments. Examples of this are individualized directional air flows and air temperatures. Directing air flow would allow treatment to be directed to reach areas in the oral mucosa most often affected by OM, which is an improvement compared to ice OC. Air temperatures could be patient controlled and thus allowing for an incremental temperature reduction of the air with individualized cooling profiles. Together these benefits of using cold air as a medium of OC could result in potentially higher compliance for patients of all ages and more effective treatment regimes.

To our knowledge, the research field of measuring the temperature of the oral mucosa in healthy volunteers after OC has rarely been researched with little evidence published in the literature. Even less research has been done in defining an appropriate bench mark temperature of the oral mucosa for optimal OC treatment and tolerability.

Overall in this pilot study, further research is needed to improve the mouthpiece and decrease the adverse events, especially related to tolerability of the IOAC device.

## Conclusion

This is the first study conducted using cold air as a medium for OC. It resulted in the average reduction of oral mucosal temperature of 10.9 °C and 14.7 °C after five and 60 min of treatment, respectively. The IOAC mouthpiece had a similar adverse profile to that of previous studies with ice, yet with a similar or better compliance. Further studies are needed in order to develop a method of delivering cold air as a cooling medium which could lower incidence of adverse events and ultimately decrease mucositis.

## Electronic supplementary material

Below is the link to the electronic supplementary material.

Supplementary file1 (PDF 518 kb)
