# Supplementary material for: Primary evaluation of an air-cooling device to reduce oral mucositis: a pilot study in healthy volunteers
Source: Med Oncol. 2020 Nov 10;37(12):110. doi: 10.1007/s12032-020-01431-4 (PMC7655586; doi:10.1007/s12032-020-01431-4)
Supplement: Supplementary file 1 — Supplementary file1 (PDF 518 kb) [file 12032_2020_1431_MOESM1_ESM.pdf]

Appendix 1

Questions on SCUBA

1. Did you manage to keep the SCUBA mouthpiece in your mouth during the entire session?

☐ Yes (go to question 4)

☐ No

2. Approximately how long did you manage to keep the SCUBA mouthpiece in your mouth?

☐ 1-20 minutes

☐ 21-30 minutes

☐ 31-40 minutes

☐ 41-50 minutes

☐ 51-59 minutes

3. Which of the following alternatives was the reason you terminated the session? More alternatives are possible.

A ☐ I was freezing

B ☐ I felt numb

C ☐ I experienced a bad taste

D ☐ I got a headache

E ☐ I experienced sensitive teeth

F ☐ My mouth started to hurt

G ☐ SCUBA fitted badly

H ☐ I felt nauseous

I ☐ I wanted to vomit

J ☐ It was difficult to swallow

K ☐ The mouthpiece was chafing

L ☐ Other.......................................

4. Was it uncomfortable to use SCUBA?

☐ No, not at all (go to question 6)

☐ No, not really

☐ Yes, a little

☐ Yes, very uncomfortable

5. If you experienced any discomfort, could you describe in which way SCUBA was uncomfortable? (More options can apply)

A ☐ I was freezing

B ☐ I got numb

C ☐ I experienced a bad taste

D ☐ I got a headache

E ☐ I experienced sensitive teeth

F ☐ My mouth started to hurt

G ☐ SCUBA fitted badly

H ☐ I felt nauseous

I ☐ I wanted to vomit

J ☐ It was difficult to swallow

K ☐ The mouthpiece was chafing

L ☐ Other......................................

6. If you were diagnosed with cancer, would you use SCUBA if it could prevent oral mucositis?

☐ Yes

☐ No

☐ Other: ………………………………………………………………………………………………

7. Other comments …………………………………………………………………………

………………………………………………………………………………………………………

………………………………………………………………………………………………………

………………………………………………………………………………………………………

………………………………………………………………………………………………………
